# Quantification of Protoporphyrin IX Accumulation in Glioblastoma Cells: A New Technique

**DOI:** 10.1155/2014/405360

**Published:** 2014-03-04

**Authors:** Johnathan E. Lawrence, Ashish S. Patel, Richard A. Rovin, Robert J. Belton, Catherine E. Bammert, Christopher J. Steele, Robert J. Winn

**Affiliations:** ^1^Upper Michigan Brain Tumor Center, Marquette, MI 49855, USA; ^2^Biology Department, Northern Michigan University, Marquette, MI 49855, USA; ^3^Purdue University, West Lafayette, IN 47906, USA; ^4^Division of Neurosurgery, Marquette General Hospital, Marquette, MI 49855, USA; ^5^School of Clinical Sciences, Northern Michigan University, Marquette, MI 49855, USA

## Abstract

*Introduction*. 5-Aminolevulinic Acid (5-ALA) is a precursor of heme synthesis. A metabolite, protoporphyrin IX (PpIX), selectively accumulates in neoplastic tissue including glioblastoma. Presurgical administration of 5-ALA forms the basis of fluorescence-guided resection (FGR) of glioblastoma (GBM) tumors. However, not all gliomas accumulate sufficient quantities of PpIX to fluoresce, thus limiting the utility of FGR. We therefore developed an assay to determine cellular and pharmacological factors that impact PpIX fluorescence in GBM. This assay takes advantage of a GBM cell line engineered to express yellow fluorescent protein. *Methods*. The human GBM cell line U87MG was transfected with a YFP expression vector. After treatment with a series of 5-ALA doses, both PpIX and YFP fluorescence were measured. The ratio of PpIX to YFP fluorescence was calculated. *Results*. YFP fluorescence permitted the quantification of cell numbers and did not interfere with 5-ALA metabolism. The PpIX/YFP fluorescence ratio provided accurate relative PpIX levels, allowing for the assessment of PpIX accumulation in tissue. *Conclusion*. Constitutive YFP expression strongly correlates with cell number and permits PpIX quantification. Absolute PpIX fluorescence alone does not provide information regarding PpIX accumulation within the cells. Our research indicates that our PpIX/YFP ratio assay may be a promising model for *in vitro* 5-ALA testing and its interactions with other compounds during FGR surgery.

## 1. Introduction

Glioblastoma multiforme (GBM) is the most common and aggressive primary malignant brain tumor. With the current standard of care, recurrence is inevitable and less than 5% of GBM patients will survive five years after diagnosis [1]. Subtotal surgical resection may contribute to recurrence as tumor initiating cells are left in place. A valuable tool to achieve gross total resection and thereby increase progression-free survival is the use of 5-Aminolevulinic Acid (5-ALA) for fluorescence-guided resection (FGR) [2]. 5-ALA is a substrate of the heme biosynthesis pathway and is the source of carbon for porphyrin synthesis [3]. Malignant gliomas metabolize 5-ALA and accumulate the fluorescent compound, protoporphyrin IX (PpIX) [4]. The presence of PpIX fluorescence within the tumor bed allows for discrimination between neoplastic and nonneoplastic cells [4]. Yet, not all brain tumors accumulate sufficient quantities of PpIX to make the use of 5-ALA advantageous during surgery [5, 6], and it is not fully understood how 5-ALA might affect other physiological processes. At this time, it is unknown if prescribed medications of tumor patients or if drugs given to patients just prior to/during surgery interfere with 5-ALA synthesis and/or PpIX accumulation. It is difficult, however, to test for the effects of these potential drug interactions during *in vitro* analyses, because fluorescence of PpIX is merely an indication of presence/absence of accumulation. PpIX fluorescence detection alone does not provide understanding about the number of metabolically active cells in an assay. This lack of information coupled with the great potential of 5-ALA in FGR suggested that a quantitative analysis of the metabolism of 5-ALA to fluorescent PpIX in tumors cells would improve the methodologies used to detect neoplasms during surgery. To accomplish this, we developed an *in vitro* assay using a YFP-tagged glioblastoma cell line. This cell line was then used to measure the rate of 5-ALA incorporation and conversion to protoporphyrin IX.

## 2. Methods

### 2.1. Cell Lines

U87MG cells (ATCC, Manassas, VA, USA) were cultured in phenol red-free Eagle's minimum essential medium (EMEM) with L-glutamine (Lonza, Portsmouth, NH, USA) and 10% fetal bovine serum (PAA, Westborough, MA, USA) at 37°C and 5% CO_2_. U87MG cells were stably transfected with the YFP expression vector (pEYFP-C1) to create U87-YFP cells using the Neon transfection system (Invitrogen, Life Technologies, Carlsbad, CA, USA) followed by selection with G418 antibiotic (Gold Biotechnology, St. Louis, MO, USA). U87MG transfection with YFP was conducted as follows: U87MG cells were harvested using Trypsin-Versene (Lonza, Portsmouth, NH, USA), counted, and resuspended in Resuspension Buffer R (Neon transfection system, Invitrogen, Life Technologies, Carlsbad, CA, USA) to a cell concentration of 5 × 10^6^ cells/mL. A cell+plasmid solution (9.5 uL resuspended cells and 0.5 uL of the pEYFP-plasmid at a concentration of 1.7 ug/mL) was prepared and 10 uL was aspirated into a Neon reaction tip. The Neon reaction tip was inserted into a Neon transfection tube filled with approximately 3 mL of Electrolytic Buffer (Neon transfection system, Invitrogen, Life Technologies, Carlsbad, CA, USA). Transfection was performed using a 1,300 pulse voltage with a 30 ms pulse width, as a single pulse. The 10 uL of transfected U87s was then pipetted into a prewarmed EMEM + 10% FBS media solution lacking antibiotics in a 96-well plate. Cells were allowed to settle and incubate for 24 hours, and then the EMEM + 10% FBS solution was replaced with media containing 0.8 mg/mL G418 antibiotic. After approximately two weeks, all of the remaining cells expressed YFP. A maintenance level of G418 (0.2 mg/mL) was then used to maintain selection pressure for YFP expressing U87s. As cells became confluent they were transferred to 24-well plates, then 6-well plates, and then finally T25 culture flasks.

### 2.2. 5-Aminolevulinic Acid Treatment

5-ALA (ACROS, Thermo Fisher Scientific, Pittsburg, PA, USA) was dissolved in phosphate buffered saline (Gibco, Life Technologies, Grand Island, NY, USA), titrated to a pH of 3, and stored at a concentration of 200 mM at −20°C. Cells were plated in black-bottom microtiter plates (Greiner Bio-One, Monroe, NC, USA) at a density of 5,000 cells per well. Cells were treated with 5-ALA solutions at 50.0, 25.0, 12.5, 6.25, 3.13, 1.56 0.78, 0.39, 0.20, and 0.10 mM. Dimethyl sulfoxide (DMSO) (Amresco, Solon, OH, USA) was added to the treatment to increase permeability of 5-ALA into the cells. Pilot studies determined that 0.5% v/v DMSO maximized 5-ALA uptake and minimized cytotoxicity. A 2% v/v of 0.1 mM ethylenediaminetetraacetic acid (EDTA) (Thermo Fisher Scientific, Pittsburg, PA, USA) was also added to reduce the availability of iron. Insulin (Sigma-Aldrich, St. Louis, MO, USA) was added at a concentration of 30 *μ*g/mL to the treatment media.

### 2.3. Fluorescence Measurement

PpIX and YFP fluorescence were measured using a Modulus Microplate Reader (Promega BioSystems, Sunnyvale, CA, USA), with a standard 525 nm excitation/580–640 nm emission filter to detect YFP fluorescence and a custom-made 405 nm excitation/580–640 nm emission filter (Promega Corporation, Madison, WI, USA) to detect PpIX fluorescence. To investigate the relationship between YFP and cell number, U87-YFP cells were counted and a standard curve was prepared by plating the cell suspension in a series dilution. To investigate relative PpIX fluorescence, baseline fluorescence values were determined at the time of treatment (hour 0) and recorded at 8, 16, 24, 32, 40, 48, 56, 64, and 72 hours after treatment. PpIX and YFP fluorescence were measured in the same plate in sequential fashion. Relative PpIX fluorescence values (PpIX fluorescent value per cell) were calculated by taking the ratio of the mean PpIX relative light units (RLU) and YFP RLU (PpIX/YFP) from each treatment well.

### 2.4. Statistical Analysis

A Pearson correlation coefficient was calculated to determine a relationship between cell number and YFP fluorescence. For PpIX florescence, individual wells from each assay were analyzed as individual values. A repeated measures analysis of variance (ANOVA) was used to analyze the differences between 5-ALA treatment concentrations and time points over 72 hours (SPSS, Version 18, IBM, Armonk, NY, USA). *Post hoc* least significant difference (LSD) analyses were used to determine statistical difference between concentrations and time points. All values are expressed as a mean ± standard error, and significant differences were determined at the 0.05 level.

## 3. Results

### 3.1. Relationship between YFP and Cell Number

To determine if yellow fluorescence emission at 580–640 nm would be a reliable mechanism to determine cell number in a well, a standard curve of U87-YFP cells was prepared. A Pearson correlation coefficient was calculated to demonstrate the relationship between YFP fluorescence and number of cells in an assay plate well. A strong positive correlation was found (*r* = 0.801 ± 0.053, *P* < 0.01), indicating a significant linear relationship between YFP RLU and the number of cells plated into a microplate well.

### 3.2. Relative PpIX Fluorescence

**Figure 1:**
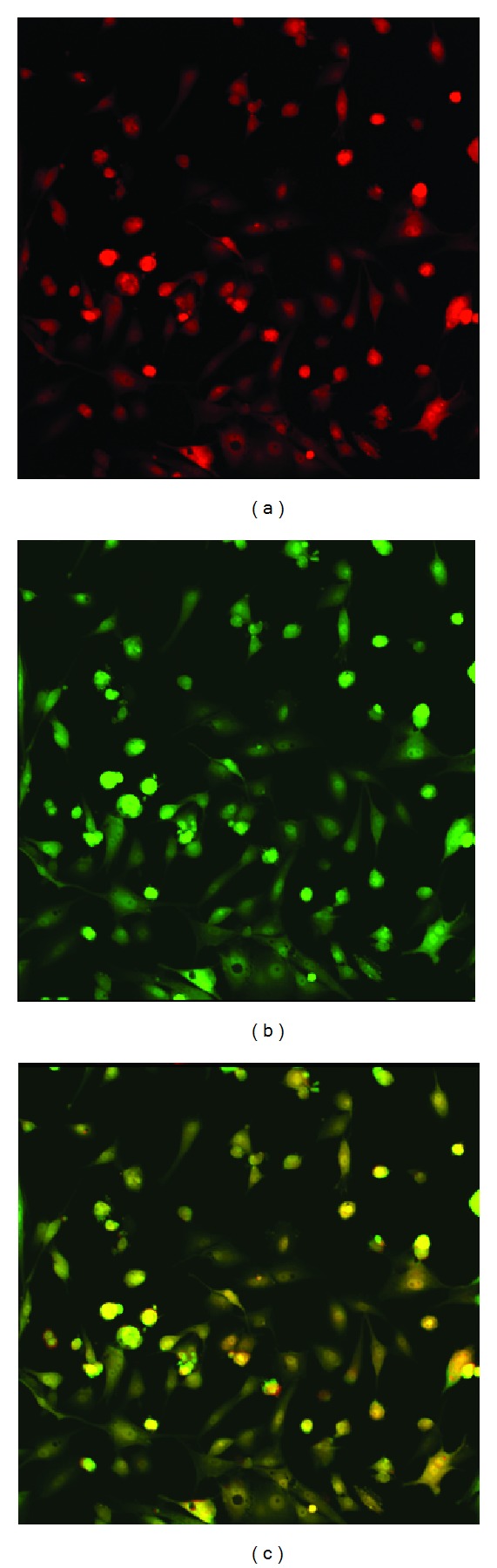
Confocal images of PpIX fluorescence (a), YFP fluorescence (b), and the overlay (c) of both for U87-YFP cells treated with 0.4 mM 5-ALA for 2 hours.

**Figure 2:**
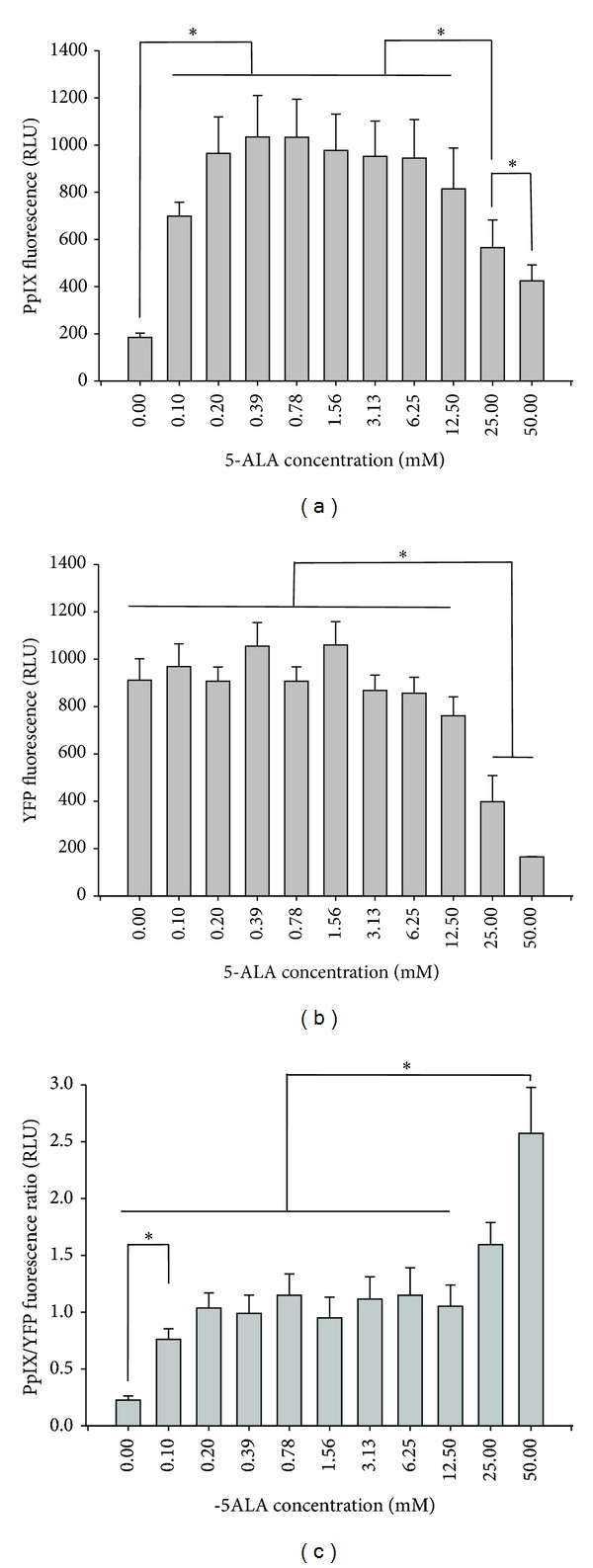
YFP and PpIX fluorescence and ANOVA main effects for each of the 5-ALA concentrations/treatments. Absolute fluorescence of PpIX (a) displays a natural bell curve for the wide range of 5-ALA treatments. YFP fluorescence (b) was not altered except for the 5-ALA treatment levels ≥12.5 mM, suggesting that elevated 5-ALA concentrations may inhibit cell growth. The bottom figure (c) represents relative PpIX accumulation (PpIX/YFP) and demonstrates that higher levels of 5-ALA allow for more PpIX production in *living* cells.

**Figure 3:**
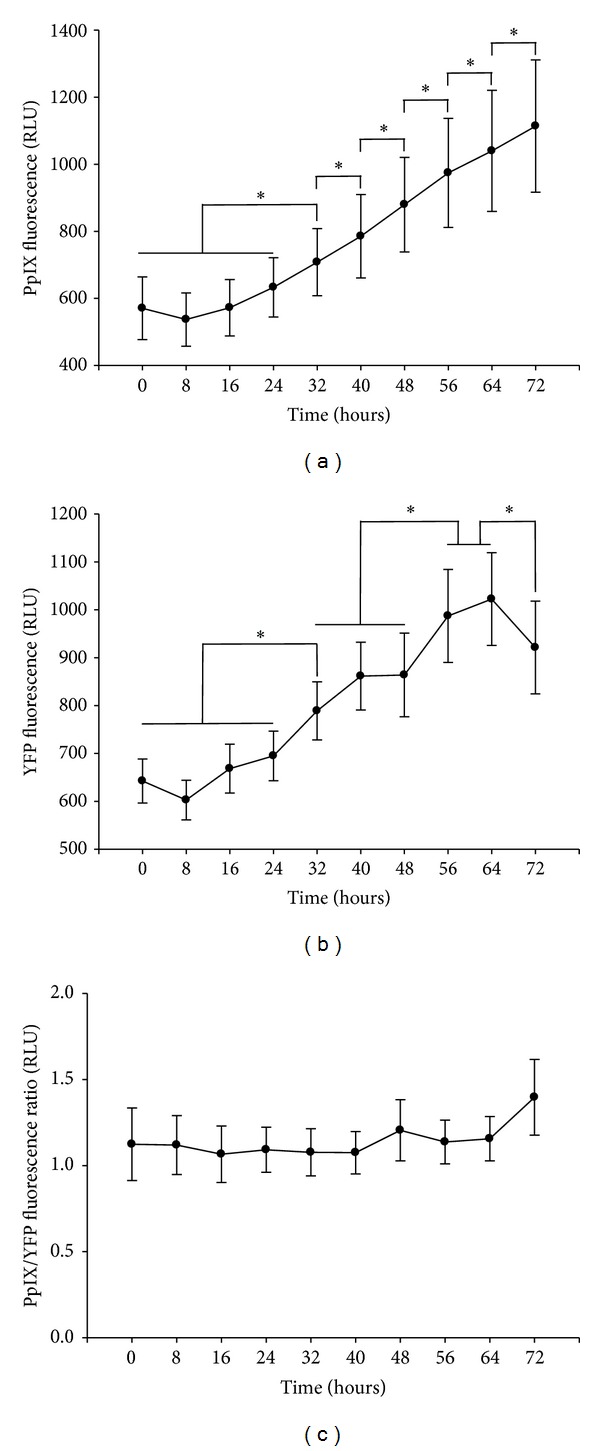
This figure displays YFP and PpIX fluorescence and ANOVA main effects for various points during a time course. Absolute fluorescence of PpIX (a) displays an increase in fluorescence over time, suggesting that cells may need more time to metabolize 5-ALA and accumulate PpIX. YFP fluorescence (b) was also increased over time, being indicative of cell growth over time. The bottom figure (c) represents relative PpIX accumulation (PpIX/YFP). The ratio demonstrates that PpIX is dependent upon the number of cells available to metabolize the 5-ALA and shows the importance of quantifying PpIX fluorescence.

After confirming YFP fluorescence and 5-ALA metabolism (PpIX fluorescence) using a confocal microscope (Figure 1), a microplate reader was used to record YFP and PpIX fluorescence and determine relative porphyrin synthesis by cells. PpIX fluorescence was compared between the eleven 5-ALA concentrations (0.0, 0.10, 0.20, 0.39, 0.78, 1.56, 3.13 6.25, 12.5, 25.0, and 50.0 mM) and the ten different time points (0, 8, 16, 24, 32, 40, 48, 56, 64, and 72 hours) using a two-way repeated measures ANOVA. When analyzing PpIX fluorescence alone, there was a significant effect found for 5-ALA concentrations (*F*(10,40) = 21.19, *P* < 0.01), time (*F*(9, 36) = 19.20, *P* < 0.01), and the interaction of 5-ALA concentration and time (*F*(90,360) = 12.54, *P* < 0.01). *Post hoc* analyses revealed the differences between concentrations (Figure 2) and time points (Figure 3).

YFP fluorescence was also compared between each 5-ALA concentration and time point. An effect was found for YFP fluorescence for 5-ALA concentration (*F*(10,40) = 27.68, *P* < 0.01), time (*F*(9,36) = 14.62, *P* < 0.01) and their interaction (*F*(90,360) = 8.41, *P* < 0.01). For differences between concentrations, see Figure 2, and for time points, see Figure 3.

Further, the ratio of PpIX fluorescence to YFP fluorescence was compared to analyze a relative amount of PpIX production within cells. For PpIX/YFP, there was an effect found for 5-ALA concentration (*F*(10,40) = 18.35, *P* < 0.01), time (*F*(9,36) = 5.87, *P* < 0.01), and their interaction (*F*(90,360) = 9.67, *P* < 0.01). For differences between concentrations, see Figure 2, and for time points, see Figure 3.

## 4. Discussion

There are several novel findings in this study: (1) constitutive YFP fluorescence strongly correlates with cell number, (2) *in vitro* PpIX fluorescence increases over time reflecting increases in cell number, (3) utilizing constitutive YFP expression in cells allows for attainable PpIX quantification *in vitro*, and (4) 5-ALA treatments ≥ 25 mM generated highest amounts of PpIX/cell while decreasing the number of viable cells. The utilization of YFP allowed for the detection of changes in cell numbers without using traditional techniques which typically damage or kill the cells—now longitudinal designs are feasible with the same assay plate. Our lab previously determined that YFP does not interfere with 5-ALA metabolism and little to no cross-talk is detectible when exciting these compounds. Thus, by making use of the PpIX/YFP fluorescence ratio, relative PpIX levels per cell are more accurately estimated and allow for the assessment of PpIX accumulation or lack thereof.

Because YFP was demonstrated to be a reliable indicator of cell number, a YFP expressing cell line provides a new model for quantifying PpIX accumulation *in vitro*. A method to quantify PpIX accumulation would allow researchers to measure of the amount of PpIX per cell. Without this quantification, PpIX fluorescence is merely an indication of absence or presence. During a time course study, for example, the number of cells in a well of an assay plate changes (increases due to cell division or decreases due to cell death). If PpIX fluorescence is recorded without accounting for the change in cell number, there is no way to determine if PpIX is accumulating within these cells. Our data suggests that higher concentrations of 5-ALA (Figure 2) may have a toxic effect in cells, yet great PpIX accumulation within living cells of the assay. These toxic effects may warrant future investigations of 5-ALA administration as a targeted therapeutic agent.

While the 5-ALA FGR method is viewed as a more efficient means for tumor resection, there are limitations. It is not fully understood how 5-ALA interacts with other physiological processes and other pharmacological agents when given systemically. 5-ALA use has been questioned because PpIX fluorescence is weak or even absent in some patients. This low PpIX fluorescence is possibly due to interactions of 5-ALA with drugs and/or other compounds circulating within tumor patients at the time of FGR surgery. Low PpIX fluorescence may also be due to the lack of individualized (patient by patient) standards regarding the presurgical administration of 5-ALA to ensure peak PpIX fluorescence. Currently, 20 mg/kg of 5-ALA per body weight is administered about 3 hours prior to surgery [7]. To our knowledge, alternative dosing strategies have not been tested for efficacy. The PpIX/YFP method may allow for dosage focused studies to be conducted.

Our data indicates that new studies designed to monitor PpIX fluorescence over time should address cell growth/death during the testing process—PpIX fluorescence *in vitro* is dependent on the number of viable cells (Figure 3). Without understanding about the number of metabolically active cells in an assay, the fluorescence of PpIX is merely an indication of presence or absence. Here, PpIX fluoresce values increased over time (Figure 3(a)); however, YFP fluorescence values also increased over time (Figure 3(b)). Further, when comparing relative PpIX fluorescence (Figure 3(c)), the amount of PpIX per cell did not change. This knowledge allows researchers to now investigate novel approaches to optimize fluorescence. Optimizing PpIX fluorescence may improve surgical outcomes and significantly increase the number of brain tumors that accumulate sufficient quantity of PpIX for FGR.

## 5. Conclusion

In summary, we showed that constitutive YFP expression strongly correlates with cell number and can be used to normalize PpIX fluorescence *in vitro*. We also showed that recording PpIX fluorescence alone does not provide information regarding accumulation of PpIX within cells, and we discovered that high levels of 5-ALA treatment reduce cell numbers during the time course warranting future investigations of 5-ALA administration as a therapeutic agent. Our PpIX/YFP ratio method is a feasible approach for *in vitro* 5-ALA analysis and may provide a new model for testing interactions of 5-ALA with other compounds circulating within tumor patients at the time of FGR surgery. These interactions may determine why some tumors fluoresce and others do not.
